# Comment for the “EpCAM-based Flow Cytometric Detection of Circulating Tumor Cells in Gallbladder Carcinoma Cases”

**DOI:** 10.31557/APJCP.2020.21.8.2179

**Published:** 2020-08

**Authors:** Gokce Gullu Amuran

**Affiliations:** *School of Medicine, Marmara University, Turkey. *

**Dear Editor**

Awasthi et al., (2017) analyzed the diagnostic role of CTCs in gallbladder cancer patients. They found that CTC quantification could be a non invasive diagnostic biomarker for the diagnosis of gallbladder carcinoma in correlation with radiological findings. Current data supports that CTCs takes place in the diagnosis, treatment and monitorizing of the cancers (Akkiprik et al., 2020; Kaigorodova et al., 2018; Lianidou,et al., 2014; Rack et al., 2014; Zhang et al., 2012). But the enrichment, isolation and enumeration approaches are crucial for CTC research. Every single day novel properties of CTCs are being found (Papadaki et al., 2018; Yu et al., 2013). Awasthi et al. used EasySep Direct Human CTC Enrichment kit (Stemcell Technologies) for the immunmagnetic negative selection of CTCs from whole blood. This kit targets hematopoietic cells and platelets for removal with antibodies recognizing CD2, CD14, CD16, CD19, CD45, CD61, CD66b and Glycophorin A. Once these cells are removed CTCs are collected for further analysis. Today we know that CTCs have different transitions states in cancer patients and they express different surface markers (Pastushenko et al., 2018; Thompson and Nagaraj, 2018). Pastushenko et al. found that hybrid mesanchimal cells express CD613 (Pastushenko et al., 2018). Since hybrid cells express CD61, Awasthi et al., (2017) missed these cells in negative selection step, more aggressive subpopulation could have been missed. Taken together Awasthi et al., (2017) should take into consideration that their results might have some shortcomings and CTC quantification for the diagnosis of gallbladder carcinoma should be re-evaluated using more precise methods.
